# Multisectoral Approach to Address Chikungunya Outbreaks Driven by Human Mobility: A Systematic Review and Meta-Analysis

**DOI:** 10.1093/infdis/jiaa500

**Published:** 2020-10-29

**Authors:** Rashad Abdul-Ghani, Florence Fouque, Mohammed A K Mahdy, Qingxia Zhong, Samira M A Al-Eryani, Abdulsamad Alkwri, John C Beier

**Affiliations:** 1 Department of Medical Parasitology, Faculty of Medicine and Health Sciences, Sana’a University, Sana’a, Yemen; 2 Tropical Disease Research Center, Faculty of Medicine and Health Sciences, University of Science and Technology, Sana’a, Yemen; 3 UNICEF/UNDP/World Bank/WHO Special Programme for Research and Training in Tropical Diseases (TDR), World Health Organization, Geneva, Switzerland; 4 Integrated Vector Management Unit, National Malaria Control Programme, Ministry of Public Health and Population, Sana’a, Yemen; 5 Department of Public Health Sciences, University of Miami Miller School of Medicine, Miami, Florida, USA

**Keywords:** chikungunya, people displacement, human mobility, multisectoral approach, outbreak

## Abstract

**Background:**

The role of human mobility in the epidemiology of emerging Aedes-transmitted viral diseases is recognized but not fully understood. The objective of this systematic review and meta-analysis was to examine how human mobility patterns are driving chikungunya outbreaks.

**Methods:**

Literature was systematically reviewed for studies on chikungunya prevalence in countries/territories with high-level evidence of human mobility-driven outbreaks, based on: (1) emergence of chikungunya outbreaks with epidemic chikungunya virus genotypes among displaced/migrant populations and their hosting communities; and (2) identification of imported index case(s) with epidemic genotypes phylogenetically related to the genotypes circulating during emerging or subsequent outbreaks.

**Results:**

The meta-analysis of extracted prevalence data revealed that a large proportion of the population in countries/territories afflicted by outbreaks is still at risk of infection during future outbreaks. On the other hand, approximately one-half of suspected chikungunya cases could be infected with other co-circulating acute febrile illnesses.

**Conclusions:**

We discussed in this paper how human mobility-driven chikungunya outbreaks can be addressed, and how the involvement of several sectors in addition to the health sector in multisectoral approaches (MSAs) is important for prevention and control of chikungunya and other Aedes-transmitted arboviral outbreaks.

The rapid spread of the chikungunya virus (CHIKV) among populations with no prior immunity is an epidemiologic feature of its current emergence as an epidemic disease [1]. On a global scale, human movements play a major role in the emergence and spread of current infectious disease outbreaks. The huge amount of human movement around the world is a threat to global health, and emerging arboviral diseases, such as chikungunya, are putting about half of the world population at risk of epidemics [2]. For instance, international tourism increased from 982 million tourists in 2011 to approximately 1.2 billion in 2015 [3]. Human movements have largely contributed to the recent expansion of chikungunya epidemics from tropical to temperate zones, such as those in Italy in 2007 and 2017 [4–6]. Evidence can be retrieved through the similarity of viral genotypes responsible for outbreaks in widely separated geographic regions. Although CHIKV genotypes belonging to different phylogenetic branches had evolved independently in historically isolated settings, some caused outbreaks linked to increased human movements in different parts of the world over recent decades [7]. For instance, the East-Central-South-African genotype is one example of the epidemic genotypes of CHIKV that emerged in East Africa and then successively moved to the Indian Ocean islands, the Indian subcontinent, Southeast Asia, Pacific Ocean islands, and more recently Europe, leading to epidemics [7]. Imported human cases among people returning from India and Indian Ocean islands to Europe and the United States have been regularly detected during chikungunya epidemics, with or without subsequent local transmission [4, 8, 9].

Travel for economic reasons has also been contributing to the global spread of chikungunya through imported cases from the tropics and subtropics to new temperate areas with nonimmune populations and competent vector mosquitoes such as *Aedes aegypti* and *Aedes albopictus*. The latter species is widely distributed and can adapt to a wide range of climates in both rural and urban environments, and can act as an epidemic vector in some settings [10, 11]. Increased human movements and trade exchanges have resulted in introducing *Ae. albopictus* into new areas, where it can establish and act as a vector for CHIKV. For instance, the introduction and dispersal of *Ae. albopictus* in the United States and Europe have been largely attributed to the trade of used tires through modern transportation and shipping [12–14]. Similarly, the reintroduction of *Ae. aegypti* from the southeastern United States to California in the summer of 2013 increases the risk of chikungunya outbreaks via imported cases [15].

There is a need for a better understanding of the impact of human mobility on the emergence of chikungunya outbreaks to design new approaches to prevent the emergence and outbreaks of arboviral diseases resulting from factors attributed to different sectors, including those related to human mobility (economic, social, and others). To show how human mobility is affecting the epidemiology of chikungunya, this study assessed the impact of human mobility on driving chikungunya outbreaks as well as how chikungunya has circulated through pooling immunoglobulin G (IgG) seroprevalence among the general population and laboratory-confirmed recent infections among suspected patients during and after outbreaks linked to human mobility with high-level evidence. A discussion follows on the best approach that can be deployed through the institutional, collaborative, and coordinate involvement of many sectors other than the health sector to prevent and control chikungunya outbreaks following displacement of people.

This review was conducted in response to a joint call for commissioned reviews from the UNICEF/UNDP/World Bank/WHO Special Programme for Research and Training in Tropical Diseases (TDR), the Swiss Agency for Development and Cooperation, the Canadian International Development and Research Centre, and the Swiss Tropical and Public Health Institute in January 2017.

## METHODS

This study was conducted in accordance with the preferred reporting items for systematic reviews and meta-analyses (PRISMA) guidelines [16], with the exception that it was not registered in a systematic review repository. However, the protocol of this commissioned review was reviewed, selected, and revised through TDR/WHO.

### Search Strategy

Published literature on emerging chikungunya autochthonous cases and outbreaks with evidence of introduction across the world was systematically reviewed. The terms “chikungunya” in [Title] and “outbreak” in [all fields] were used to search PubMed and Scopus for journal articles [publication type] published from 1 January 2004 to 31 December 2017, with no language restrictions. Because the emergence of the new East/Central/South African genotype of the virus on Lamu Island of Kenya in 2004 was the takeoff point of the recent CHIKV circulation and outbreaks around the world [17], this year was used as the lower time boundary of our search. The search was then refined by limiting the results to studies in [Humans]. Citations of the retrieved publications with their abstracts were imported into EndNote X8 for Windows (Thompson Reuters). Reference lists of retrieved studies were also searched for any relevant articles not identified from database searching. Titles and abstracts of the selected studies were screened to remove duplicate or irrelevant publications.

### Identification of Chikungunya Outbreaks Linked to Human Mobility with High-Level Evidence

High-level evidence on the role of human mobility as a driver for chikungunya outbreaks in afflicted countries (Supplementary Table 1) was determined based on 2 criteria: (1) emergence of chikungunya outbreaks with epidemic CHIKV genotypes among displaced/migrant populations and their hosting communities, and (2) identification of imported index case(s) with epidemic genotypes phylogenetically related to the genotypes circulating during emerging or subsequent outbreaks.

### Study Selection and Assessment of Chikungunya Circulation in Outbreaks Linked to Human Mobility

Full-text publications on cross-sectional studies reporting chikungunya IgG seroprevalence or prevalence of laboratory-confirmed recent infections (by IgM serology, reverse transcription-polymerase chain reaction [RT-PCR] and/or virus isolation) in countries with chikungunya outbreaks linked to human mobility with high-level evidence were selected (Supplementary Table 2). Studies reporting IgG seroprevalence among the general population of both sexes and any age were selected for pooling the past exposure to infection after chikungunya outbreaks. On the other hand, studies conducted among suspected patients were selected for pooling the prevalence of laboratory-confirmed recent infections. Outbreak investigations were included if prevalence outcomes could be calculated. Observational studies other than cross-sectional ones, interventional and clinical studies, and cross-sectional studies with unclear design or inappropriate presentation of results or on imported chikungunya in travelers not identified as index cases for outbreak emergence were excluded.

### Data Extraction and Analysis

Prevalence data were extracted from the full-text articles of eligible studies (Supplementary Table 2). Extracted data were meta-analyzed using MetaXL software, version 5.3 (EpiGear International). The primary outcomes of pooled IgG seroprevalence among the general population and pooled prevalence of laboratory-confirmed recent infections among suspected patients were calculated with their 95% confidence intervals (95% CIs). Because of the expected differences in the study samples, designs, and populations, meta-analyses to determine pooled outcomes were performed using a random-effects model [18]. The proportion of total variation in study estimates attributed to heterogeneity was measured using the *I*^*2*^ statistic [19], where *I*^*2*^ > 50% indicates high heterogeneity. In addition, forest plots were used to show the random effects of pooled outcomes.

### Assessment of Risk of Bias

The risk of bias in the selection of cross-sectional studies was assessed using the appraisal checklist of the Joanna Briggs Institute for studies reporting prevalence data [20], with slight modification (Supplementary Table 3). Six criteria were adopted in the appraisal of the quality and assessment of the risk of bias: study design, study population, study setting, sampling method, method(s) of IgG detection, and method(s) of confirming recent infection. Each study was rated for risk of bias by scoring 5 of the above 6 criteria, each with 2 score options (2 or 1). Accordingly, studies for pooling IgG seroprevalence were not assessed against the criterion “Methods of confirming recent infection” and studies for pooling the prevalence of laboratory-confirmed recent infection were not assessed against the criterion “Method(s) of IgG detection.” Only studies scoring high for quality and low for risk of bias (total score ≥ 9) were selected for inclusion in the meta-analysis.

## RESULTS

### Countries Identified with Human Mobility-Driven Chikungunya with High-Level Evidence

Supplementary Table 1 shows the countries/territories with outbreaks linked to human mobility with high-level evidence by identifying imported index case(s) infected by epidemic genotypes of CHIKV followed by outbreaks or autochthonous transmission of phylogenetically related genotypes. Fourteen countries from 5 WHO regions were identified with outbreaks linked to human mobility with high-level evidence.

### Characteristics of Studies for Pooling IgG Seroprevalence and Confirmed Cases

**Figure 1.**
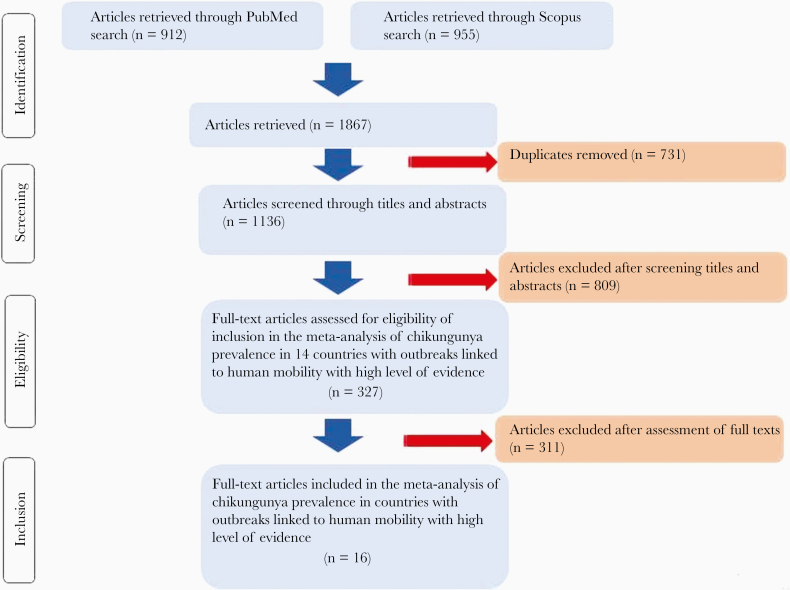
Flowchart of study identification and selection for inclusion in the meta-analysis.

**Figure 2.**
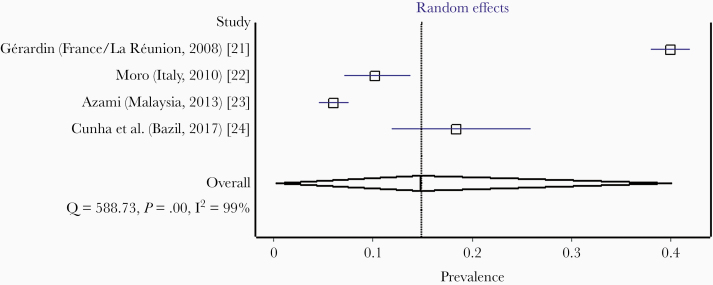
Forest plot showing a random-effects meta-analysis of pooled chikungunya IgG seroprevalence.

**Figure 3.**
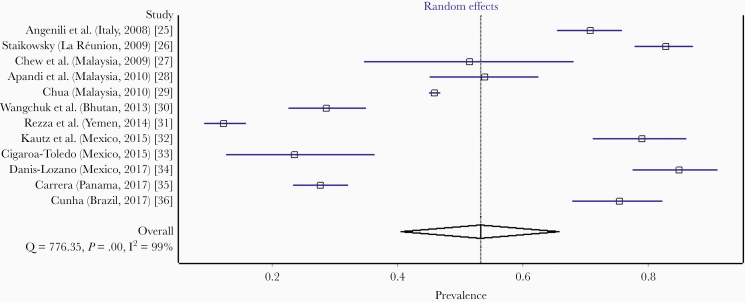
Forest plot showing a random-effects meta-analysis of pooled prevalence of laboratory-confirmed recent chikungunya infection.

Searching using PubMed and Scopus yielded 1867 records (Figure 1). Of these, 16 publications from 8 countries [21–36] (Supplementary Table 2) were included in the meta-analysis of human mobility-driven chikungunya outbreaks. Four studies from La Réunion, Italy, Malaysia, and Brazil were selected for pooling IgG seroprevalence. These studies comprised 3814 individuals sampled from the general population after outbreaks (Table 1 and Figure 2). Twelve studies from Italy, La Réunion, Malaysia, Bhutan, Yemen, Mexico, Panama, and Brazil were selected for pooling the prevalence of laboratory-confirmed recent infections. These studies comprised 15 944 individuals sampled from suspected patients during outbreaks (Table 2 and Figure 3).

**Table 1.** Pooled Anti-CHIKV IgG Seroprevalence Following Emerging Chikungunya Outbreaks Linked to Human Mobility With High-Level Evidence (2004–2017)

First Author (Country or Territory, Year of Publication)	Total Examined	Number Infected	Prevalence, % (95% CI)	Weight, %	Ref.
Gérardin (La Réunion, 2008)	2424	967	39.9 (38.0–41.9)	25.2	[21]
Moro (Italy, 2010)	325	33	10.2 (7.1–13.7)	25.0	[22]
Azami (Malaysia, 2013)	945	56	5.9 (4.5–7.5)	25.2	[23]
Cunha (Brazil, 2017)	120	22	18.3 (11.9–25.8)	24.6	[24]
Total (random effects)	3814	1078	14.8 (.3–40.0)	100.0	

Abbreviations: CHIKV, chikungunya virus; CI, confidence interval.

**Table 2.** Pooled Prevalence of Laboratory-Confirmed Recent Infection With Chikungunya During Emerging Outbreaks Linked to Human Mobility With High-Level Evidence (2004–2017)

First Author (Country or Territory, Year of Publication)	Total Examined	Number Infected	Prevalence, % (95% CI)	Weight (%)	Ref.
Angelini (Italy, 2008)	307	217	70.7 (65.5–75.7)	8.5	[25]
Staikowsky (La Réunion, 2009)	266	220	82.7 (77.9–87.0)	8.5	[26]
Chew (Malaysia, 2009)	35	18	51.4 (34.8–67.9)	7.6	[27]
Apandi (Malaysia, 2010)	130	70	53.8 (45.2–62.4)	8.3	[28]
Chua (Malaysia, 2010)	13759	6314	45.9 (45.1–46.7)	8.7	[29]
Wangchuk (Bhutan, 2013)	210	60	28.6 (22.6–34.9)	8.5	[30]
Rezza (Yemen, 2014)	400	49	12.3 (9.2–15.7)	8.6	[31]
Kautz (Mexico, 2015)	119	94	79.0 (71.2–85.9)	8.3	[32]
Cigarroa-Toledo (Mexico, 2016)	51	12	23.5 (12.8–36.3)	7.9	[33]
Danis-Lozano (Mexico, 2017)	112	95	84.8 (77.5–90.9)	8.3	[34]
Carrera (Panama, 2017)	413	114	27.6 (23.4–32.0)	8.6	[35]
Cunha (Brazil, 2017)	142	107	75.4 (67.9–82.1)	8.4	[36]
Total (random effects)	15 944	7370	53.2 (40.5–65.7)	100.0	

Recent infection was defined as that confirmed by CHIKV RNA by reverse transcription-polymerase chain reaction (RT-PCR) and/or virus isolation in culture and/or IgM serology.

Abbreviations: CHIKV, chikungunya virus; CI, confidence interval.

### Pooled IgG Seroprevalence

The pooled seroprevalence of anti-CHIKV IgG among asymptomatic populations following human-mobility driven outbreaks in the selected countries was 14.8% (95% CI, .3–40.0; *I*^*2*^* = *99.0%) based on the random-effects meta-analysis of 4 studies (Table 1) [21–24]. However, the forest-plot analysis and high *I*^*2*^ value (Figure 2) show significant heterogeneity in IgG seroprevalence among the populations, which was more pronounced between the different countries/studies but was lower within the same country/study, where it ranged from < 10% to approximately 40.0%. Such heterogeneity may represent infections with more or less virulent CHIKV strains and must be considered when estimating the real burden of the epidemic in the affected populations. Nevertheless, these results demonstrate that in most of the countries with high-level evidence of human mobility-driven outbreaks, only a small proportion (around 15%) of the population had been infected and acquired some degree of immunity.

### Pooled Prevalence of Laboratory-Confirmed Chikungunya

The pooled prevalence of laboratory-confirmed recent infections among suspected patients during emerging outbreaks linked to people displacement or other human movements was 53.2% (95% CI, 40.5–65.7; *I*^*2*^ =  99.0%) based on a random-effects meta-analysis of 12 studies [25–36] (Table 2). The forest-plot analysis and *I*^*2*^ value (Figure 3) show a significant heterogeneity between studies and within each study and within each country/study.

## DISCUSSION

The findings of this study identified chikungunya outbreaks linked to human mobility with high-level evidence across 5 WHO regions, clearly demonstrating that chikungunya outbreaks driven by human mobility are occurring worldwide. The circulation of epidemic genotypes of CHIKV highlights the major role of human mobility patterns in disseminating chikungunya, as an example of emerging arboviral diseases, to nonendemic areas or areas endemic for different genotypes of the virus at the global level. This is particularly important with the increasing trend in global population mobility because of globalization, extensive trade, and travel events. A pooled IgG seroprevalence of approximately 15% was observed for chikungunya among individuals sampled from the general population from countries with outbreaks linked to human mobility with high-level evidence. This result shows that around 85% of the populations in the countries included are still nonimmune and at risk of infection for future outbreaks. Thus, the commonly accepted idea that an outbreak is creating a group-immunity that may protect the susceptible population is in question, and, in the same way, the interval between outbreaks may be shorter than usually thought.

Laboratory-confirmed recent chikungunya linked to human mobility was prevalent among approximately one-half of suspected patients, with variability across different countries. For instance, laboratory-confirmed recent infections ranged from as low as less than 20% among suspected cases in Yemen [31] to about 85% of suspected cases in Mexico [34]. Therefore, the symptoms in about half of suspected patients could not be attributed to chikungunya but probably to other acute febrile illnesses cocirculating at the times of emerging chikungunya outbreaks, leading to overdiagnosis of cases through the clinical symptoms because only about half of the suspected cases were confirmed. This shows that during a chikungunya outbreak linked to human mobility, about half of the febrile cases may be due to other causes. As a result, the differential diagnosis of chikungunya from other cocirculating acute febrile illnesses during emerging outbreaks is strongly recommended.

The impact of human mobility on chikungunya outbreaks highlights the pressing need for a good surveillance and reporting system to notify health authorities about any clustering of chikungunya. A standardized case definition should be used for reporting and notification of chikungunya cases [37] resulting from people displacement or other human mobility patterns, notably the algorithm set by the Joint Mission of the European Centre for Disease Prevention and Control and WHO [6], and the algorithm developed and recommended by the US Centers for Diseases Prevention and Control [38]. In areas of conflicts or disasters with frequent people displacement, the adoption of active syndromic surveillance could help to detect outbreaks earlier in support of traditional passive surveillance.

Furthermore, human mobility-driven outbreaks of chikungunya emphasize the requirement of multisectoral approaches (MSAs) to the prevention and control of this disease. The lessons learned from the reviewed publications on earlier outbreaks show some interesting approaches. In La Réunion, during the campaign against chikungunya from 2005 to 2006 [39, 40], the French health authorities established a task force to develop an interdisciplinary approach to control the outbreak together with the adoption of the recommended public health measures [41]. This task force included physicians, specialists in public health and social sciences, virologists, immunologists, entomologists, and pathologists besides other sectors such as tourism, economy, legislation, and agriculture.

MSAs are recommended by the WHO Global Strategic Framework on Integrated Vector Management (IVM) and the Global Vector Control Response 2017–2030 (GVCR) [2, 42], and publications have reported promising case studies. Among the main activities that must be included in a MSA, the GVCR prioritizes the establishment of a national interministerial task force for multisectoral coordination of vector control activities and intra- and intersectoral collaborations are placed as 1 of the 4 pillars of the framework. A recently published integrated *Aedes* management framework was also developed to improve control of *Aedes*-borne arboviral diseases [43]. Moreover, the recently released TDR guidance for multisectoral prevention and control of vector-borne diseases [44] provides an overview of the paths and elements that are needed to address disease transmission. In situations where sizable populations are displaced, the conditions for vector multiplication are often very favorable. Therefore, the surveillance of the vector is essential. Successful IVM strategies were adopted during emerging outbreaks in some Caribbean territories [45] and an IVM strategy adapted from the WHO strategic framework [40], particularly in evacuation centers, can be adapted.

Within the context of emerging chikungunya outbreaks due to people displacement, and according to the IVM framework [42], the major technical and multisectoral elements must include: (1) outbreak risk assessment, (2) communication, (3) mobilization and education of displaced populations, (4) mobilization and involvement of stakeholders, (5) rapid notification and strengthened surveillance system, and (6) vector control.

Risk assessment of vector-borne diseases outbreaks and communication in situations of people displacement into new environments following conflicts or disasters should involve not only the health sector but also the education and environment sectors. This assessment can be performed according to the standard WHO guidelines for the rapid risk assessment of acute public health events [46]. Risk perceptions, attitudes, and knowledge of chikungunya among the general public and health professionals can be lower in areas that have not experienced an outbreak [47]. Proactive education and risk communication to displaced or forcedly migrant people and recipient communities is thus a strong necessity to enhance preparedness and response of local and international stakeholders.

A robust coordination pathway and the institutionalization of coordination mechanisms will ensure the meaningful and effective participation of all relevant stakeholders [44]. The sectors involved in such MSAs can span from education, mass media, and social media at all levels (local authorities, community leaders, healthcare workers, teachers, etc.) to security and defense that can facilitate the tasks of surveillance, prevention, and control and to finance and human resources that can help in securing and managing the financial and human resources for implementing the activities related to the containment of outbreaks.

Although this study is limited by the retrieval of peer-reviewed data from 2 databases, it provides high-level evidence on human mobility-driven chikungunya outbreaks, showing that a high proportion of nonimmune people are exposed to the risk of future outbreaks and that case reporting is not optimal in many countries unless laboratory-confirmed. Further studies are thus required to assess the impact of human mobility on chikungunya outbreaks on morbidity and mortality. It would also be very interesting to investigate how much of the chikungunya transmission is not only due to human mobility but also to local emergence. Moreover, the criteria used for assessing the level of evidence may not address all possible risks of bias. Therefore, the findings of this study should be interpreted cautiously in the context of the criteria used for selecting the studies.

## CONCLUSION

This study demonstrates the impact of human mobility on the emergence of chikungunya outbreaks, with pooled anti-CHIKV IgG seroprevalence of approximately 15% across different countries/territories with outbreaks linked to human mobility with high-level evidence. Accordingly, most populations in countries/territories afflicted with such outbreaks are still at high risk of infections and human mobility is still a potential driver for future outbreaks. These results also probably provide an indication of the status of other arboviral diseases transmitted by the same mosquito species. On the other hand, chikungunya is not the only cause of febrile episodes during the emergence of chikungunya outbreaks, as revealed by the prevalence of laboratory-confirmed recent infections among about half of suspected patients. To address the challenge of human-mobility driven emergence of outbreaks, there is a need for MSAs involving partnerships and collaborations at national, regional, and global levels. The huge amount of human movement becomes increasingly demanding for the containment of *Aedes*-transmitted arboviral outbreaks. Some lessons learned from successful MSAs should be evaluated for replication in situations of potential emergence of outbreaks due to human mobility patterns such as people displacement. Furthermore, evidence should be derived from a wider range of socioeconomic, cultural, and geographic contexts involving different countries and regions.

## Supplementary Data

Supplementary materials are available at *The Journal of Infectious Diseases* online. Consisting of data provided by the authors to benefit the reader, the posted materials are not copyedited and are the sole responsibility of the authors, so questions or comments should be addressed to the corresponding author.

Notes

jiaa500_suppl_Supplementary_Table_1

jiaa500_suppl_Supplementary_Table_2

jiaa500_suppl_Supplementary_Table_3

## Notes

***Acknowledgments.*** We thank Audrey Lenhart, Center for Global Health/Division of Parasitic Diseases and Malaria/Entomology Branch, US Centers for Disease Control and Prevention for her invaluable comments during the preparation of this commissioned review; and André B. B. Wilke, Department of Public Health Sciences, University of Miami Miller School of Medicine for his help during the preparation of the manuscript.

***Financial support.*** This work was supported by the UNICEF/UNDP/World Bank/WHO Special Programme for Research and Training in Tropical Diseases (WHO grant number 2017/ 721320-0).

***Supplement sponsorship.*** This Supplement is sponsored by TDR: the Special Programme for Research and Training in Tropical Diseases co-sponsored by the United Nations Children’s Fund (UNICEF), the United Nations Development Programme (UNDP), the World Bank and the World Health Organization (WHO).

***Potential conflicts of interest.*** All authors: No reported conflicts of interest. All authors have submitted the ICMJE Form for Disclosure of Potential Conflicts of Interest. Conflicts that the editors consider relevant to the content of the manuscript have been disclosed.
