# Supplementary material for: Multisectoral Approach to Address Chikungunya Outbreaks Driven by Human Mobility: A Systematic Review and Meta-Analysis
Source: J Infect Dis. 2020 Oct 29;222(Suppl 8):S709–16. doi: 10.1093/infdis/jiaa500 (PMC7594244; doi:10.1093/infdis/jiaa500)
Supplement: jiaa500_suppl_Supplementary_Table_1 [file jiaa500_suppl_supplementary_table_1.docx]

**Supplementary Table 1.** Countries with emerging chikungunya outbreaks linked to human mobility with high-level evidence based on the identification of the index imported case(s) with epidemic CHIKV genotypes phylogenetically related to the circulating genotypes during outbreaks (2004–2017)

| **Country** | **Start year of emergence** | **Imported index case(s) or an infected displaced/migrant population category identified with epidemic genotypes of the virus** | **CHIKV genotype(s) circulating during the outbreaks*** | **Ref.** |
| --- | --- | --- | --- | --- |
| French Overseas Territory (La Réunion ) | 2005 | - Imported index cases from Comoros | ECSA | [1–3] |
| Malaysia | 2006 | - Indian female patient returning from Chennai, Tamil Nadu to Batu Gajah, Perak in August 2006 - Indian male patient returning from Paramakudi -Tamil Naidu to Kinta, Perak in Nov. 2006 | ECSA (& Asian genotype by one study) | [4–9] |
| Singapore | 2006 | - Confirmation of infection among returning Indian and Malaysian travelers visiting friends and relatives - Emergence of an outbreak in an industrial area with foreign migrant workers from epidemic countries and confirmation of infection among a high proportion of them | ECSA | [10–14] |
| Italy | 2007 | - Index case is an Indian man from Kerala State | ECSA | [15–18] |
| France | 2010 | - Index cases were travelers returning from Rajasthan, India and from Cameroon | ECSA | [19–21] |
| New Caledonia | 2011 | - Two imported index cases returning from Indonesia | Asian | [22] |
| Yemen | 2011 | - Eritrean refugees (detection of the ECSA genotype of the virus in *Aedes aegypti* in their camps) | ECSA | [23–25] |
| Bhutan | 2012 | - Index case was a traveler from West Bengal, India | ECSA | [26] |
| Dominica | 2014 | - Index case with a recent travel history to Saint Martin and confirmed in Dec. 2013 | Asian | [27–29] |
| Panama | 2014 | - Two imported index cases before the emergence of autochthonous cases: an infected man returning from Haiti and an infected woman returning from the Dominican Republic | Asian | [30, 31] |
| Brazil | 2014 | - A Brazilian citizen living in Luanda of Angola and returning to visit his family - Two index cases preceding the emergence of the Asian genotype in Amapa state (returning from the Dominican Republic and Guadeloupe). - One index case preceding the emergence of the ECSA genotype in Bahia state was a traveler returning from Angola. | ECSA & Asian | [32–37] |
| Mexico | 2014 | - Index case was an infected sportswoman from the Caribbean island of Antigua and Barbuda to Jalisco, Mexico in May 2014. | Asian | [38–42] |
| French Polynesia | 2014 | - Index case was a woman traveler returning from Guadeloupe in May 2014. | Asian | [43, 44] |

* Phylogenetic evidence was based on the identification of “epidemic” genotypes during the ongoing outbreak(s) but not necessarily reported by all studies conducted during or post outbreaks; CHIKV, chikungunya virus; ECSA, East/Central/South African genotype.

**References**

1. Quatresous I, The Investigation Group. E-alert 27 January: Chikungunya outbreak in Réunion, a French ‘overseas département’. Euro Surveill **2006**; 11:E060202.1.
2. Kariuki Njenga M, Nderitu L, Ledermann JP, et al. Tracking epidemic Chikungunya virus into the Indian Ocean from East Africa. J Gen Virol **2008**; 89:2754–60.
3. Schuffenecker I, Iteman I, Michault A, et al. Genome microevolution of chikungunya viruses causing the Indian Ocean outbreak. PLoS Med **2006**; 3:e263.
4. Soon YY, Junaidi I, Kumarasamy V, Chem YK, Juliana R, Chua KB. Chikungunya virus of Central/East African genotype detected in Malaysia. Med J Malaysia **2007**; 62:214–7.
5. Noridah O, Paranthaman V, Nayar SK, et al. Outbreak of chikungunya due to virus of Central/East African genotype in Malaysia. Med J Malaysia **2007**; 62:323–8.
6. Chem YK, Zainah S, Berendam SJ, Rogayah TA, Khairul AH, Chua KB. Molecular epidemiology of chikungunya virus in Malaysia since its first emergence in 1998. Med J Malaysia **2010**; 65:31–5.
7. Sam IC, Chan YF, Chan SY, et al. Chikungunya virus of Asian and Central/East African genotypes in Malaysia. J Clin Virol **2009**; 46:180–3.
8. Apandi Y, Lau SK, Izmawati N, et al. Identification of chikungunya virus strains circulating in Kelantan, Malaysia in 2009. Southeast Asian J Trop Med Public Health **2010**; 41:1374–80.
9. Yusoff AF, Mustafa AN, Husaain HM, et al. The assessment of risk factors for the Central/East African genotype of chikungunya virus infections in the state of Kelantan: a case control study in Malaysia. BMC Infect Dis **2013**; 13:211.
10. Shu PY, Yang CF, Su CL, et al. Two imported chikungunya cases, Taiwan. Emerg Infect Dis **2008**; 14:1326–7.
11. Leo YS, Chow AL, Tan LK, Lye DC, Lin L, Ng LC. Chikungunya outbreak, Singapore, 2008. Emerg Infect Dis **2009**; 15:836–7.
12. Ng LC, Tan LK, Tan CH, et al. Entomologic and virologic investigation of chikungunya, Singapore. Emerg Infect Dis **2009**; 15:1243–9.
13. Lim PL, Oh HM, Ooi EE. Chikungunya in Singapore: imported cases among travelers visiting friends and relatives. J Travel Med **2009**; 16:289–91.
14. Win MK, Chow A, Dimatatac F, Go CJ, Leo YS. Chikungunya fever in Singapore: acute clinical and laboratory features, and factors associated with persistent arthralgia. J Clin Virol **2010**; 49:111–4.
15. Angelini R, Finarelli AC, Angelini P, et al. An outbreak of chikungunya fever in the province of Ravenna, Italy. Euro Surveill **2007**; 12:E070906 1.
16. Rezza G, Nicoletti L, Angelini R, et al. Infection with chikungunya virus in Italy: an outbreak in a temperate region. Lancet **2007**; 370:1840–6.
17. Venturi G, Di Luca M, Fortuna C, et al. Detection of a chikungunya outbreak in Central Italy, August to September 2017. Euro Surveill **2017**; 22.
18. Carletti F, Marsella P, Colavita F, et al. Full-length genome sequence of a chikungunya virus isolate from the 2017 autochthonous outbreak, Lazio Region, Italy. Genome Announc **2017**; 5.
19. Grandadam M, Caro V, Plumet S, et al. Chikungunya virus, southeastern France. Emerg Infect Dis **2011**; 17:910–3.
20. Delisle E, Rousseau C, Broche B, et al. Chikungunya outbreak in Montpellier, France, September to October 2014. Euro Surveill **2015**; 20:21108.
21. Calba C, Guerbois-Galla M, Franke F, et al. Preliminary report of an autochthonous chikungunya outbreak in France, July to September 2017. Euro Surveill **2017**; 22.
22. Alibert A, Pfannstiel A, Grangeon JP. Chikungunya outbreak in New Caledonia in 2011, Status report as at 22 August 2011. Inform’Action **2011**; 34:3–9.
23. Zayed A, Awash AA, Esmail MA, et al. Detection of chikungunya virus in Aedes aegypti during 2011 outbreak in Al Hodayda, Yemen. Acta Trop **2012**; 123:62–6.
24. Fahmy NT, Klena JD, Mohamed AS, Zayed A, Villinski JT. Complete genome sequence of chikungunya virus isolated from an *Aedes aegypti* mosquito during an outbreak in Yemen, 2011. Genome Announc **2015**; 3.
25. Ciccozzi M, Lo Presti A, Cella E, et al. Phylogeny of dengue and chikungunya viruses in Al Hudayda governorate, Yemen. Infect Genet Evol **2014**; 27:395–401.
26. Wangchuk S, Chinnawirotpisan P, Dorji T, et al. Chikungunya fever outbreak, Bhutan, 2012. Emerg Infect Dis **2013**; 19:1681–4.
27. Ahmed S, Francis L, Ricketts RP, Christian T, Polson-Edwards K, Olowokure B. Chikungunya virus outbreak, Dominica, 2014. Emerg Infect Dis **2015**; 21:909–11.
28. Imai K, Nakayama E, Maeda T, et al. Chikungunya fever in Japan imported from the Caribbean islands. Jpn J Infect Dis **2016**; 69:151–3.
29. Sahadeo NSD, Allicock OM, De Salazar PM, et al. Understanding the evolution and spread of chikungunya virus in the Americas using complete genome sequences. Virus Evol **2017**; 3:vex010.
30. Diaz Y, Carrera JP, Cerezo L, et al. Chikungunya virus infection: first detection of imported and autochthonous cases in Panama. Am J Trop Med Hyg **2015**; 92:482–5.
31. Carrera JP, Diaz Y, Denis B, et al. Unusual pattern of chikungunya virus epidemic in the Americas, the Panamanian experience. PLoS Negl Trop Dis **2017**; 11:e0005338.
32. Teixeira MG, Andrade AM, Costa Mda C, et al. East/Central/South African genotype chikungunya virus, Brazil, 2014. Emerg Infect Dis **2015**; 21:906–7.
33. Nunes MR, Faria NR, de Vasconcelos JM, et al. Emergence and potential for spread of chikungunya virus in Brazil. BMC Med **2015**; 13:102.
34. Cunha MS, Cruz NVG, Schnellrath LC, et al. Autochthonous transmission of East/Central/South African genotype chikungunya virus, Brazil. Emerg Infect Dis **2017**; 23:1737–9.
35. Souza TM, Azeredo EL, Badolato-Correa J, et al. First report of the East-Central South African genotype of chikungunya virus in Rio de Janeiro, Brazil. PLoS Curr **2017**; 9.
36. Charlys da Costa A, Theze J, Komninakis SCV, et al. Spread of chikungunya Virus East/Central/South African genotype in northeast Brazil. Emerg Infect Dis **2017**; 23:1742–4.
37. Cunha MDP, Santos CAD, Neto DFL, et al. Outbreak of chikungunya virus in a vulnerable population of Sergipe, Brazil - A molecular and serological survey. J Clin Virol **2017**; 97:44–9.
38. Rivera-Avila RC. Chikungunya fever in Mexico: confirmed case and notes on the epidemiologic response. Salud Publica Mex **2014**; 56:402–4.
39. Diaz-Quinonez JA, Ortiz-Alcantara J, Fragoso-Fonseca DE, et al. Complete genome sequences of chikungunya virus strains isolated in Mexico: first detection of imported and autochthonous cases. Genome Announc **2015**; 3.
40. Diaz-Quinonez JA, Escobar-Escamilla N, Ortiz-Alcantara J, et al. Identification of Asian genotype of chikungunya virus isolated in Mexico. Virus Genes **2016**; 52:127–9.
41. Kautz TF, Diaz-Gonzalez EE, Erasmus JH, et al. Chikungunya virus as cause of febrile illness outbreak, Chiapas, Mexico, 2014. Emerg Infect Dis **2015**; 21:2070–3.
42. Cigarroa-Toledo N, Blitvich BJ, Cetina-Trejo RC, et al. Chikungunya virus in febrile humans and *Aedes aegypti* mosquitoes, Yucatan, Mexico. Emerg Infect Dis **2016**; 22:1804–7.
43. Nhan TX, Claverie A, Roche C, et al. Chikungunya virus imported into French Polynesia, 2014. Emerg Infect Dis **2014**; 20:1773–4.
44. Aubry M, Teissier A, Roche C, et al. Chikungunya outbreak, French Polynesia, 2014. Emerg Infect Dis **2015**; 21:724–6.
