# Supplementary material for: Multisectoral Approach to Address Chikungunya Outbreaks Driven by Human Mobility: A Systematic Review and Meta-Analysis
Source: J Infect Dis. 2020 Oct 29;222(Suppl 8):S709–16. doi: 10.1093/infdis/jiaa500 (PMC7594244; doi:10.1093/infdis/jiaa500)
Supplement: jiaa500_suppl_Supplementary_Table_2 [file jiaa500_suppl_supplementary_table_2.docx]

**Supplementary Table 2.** Characteristics of studies included in the meta-analysis of chikungunya prevalence from countries with outbreaks linked to human mobility with high-level evidence (2004–2017)

| **Study**  **#** | **First author** (country or  territory, year of publication) | **Study design/ sampling strategy** | **Study setting** | **Study population** | **Sample size*** | **Study period** | **Detection method(s)** | **Ref.** |
| --- | --- | --- | --- | --- | --- | --- | --- | --- |
| **Characteristics of studies meta-analyzed for pooling IgG seroprevalence among the general population** | | | | | | | | |
| 1 | Gérardin (La Réunion, 2008) | Household-based, ross-sectional/ two-stage, random | La Réunion island | General population | 2424 | Aug. to Oct. 2006 | ELISA | [21] |
| 2 | Moro (Italy, 2010) | Cross-sectional/ systematic random | Castiglione di Cervia village, Emilia-Romagna Region | General population | 307 | 2007 (3-5 months post outbreak) | IFAT | [22] |
| 3 | Azami (Malaysia, 2013) | Cross-sectional | Kuala Lumpur, Selangor, Pahang and Negeri Sembilan) | Healthy adults | 945 | 2009 | ELISA | [23] |
| 4 | Cunha (Brazil, 2017) | Household-based, cross-sectional/ random clustered | Chapada district; Bahia state in the northeast  (total pop.:  2303) | General population | 120 | April 2016 (after the first outbreak) | ELISA | [24] |
| **Characteristics of studies meta-analyzed for pooling the prevalence of laboratory-confirmed recent chikungunya among suspected patients** | | | | | | | | |
| 1 | Angelini (Italy, 2008) | Outbreak investigation/ all suspected patients in the study setting were sampled | Emilia-Romagna Region | Patients with suspected chikungunya | 377 | Jul. to Sept. 2007 | RT-PCR | [25] |
| 2 | Staikowsky (La Réunion, 2009) | Outbreak investigation/ consecutive sample of patients | La Réunion | Patients with febrile arthralgia suspected of having chikungunya | 266 | 1^st^ Mar. to 31^st^ May 2006 | RT-PCR, IgM-ELISA | [26] |
| 3 | Chew (Malaysia, 2009) | Hospital-based, cross-sectional/ convenience sampling | Johor Bahru, Johor State | Patients aged >12 years with suspected chikungunya | 35 | April to Aug. 2008 (during the outbreak) | RT-PCR, virus isolation, IgM-ELISA | [27] |
| 4 | Apandi (Malaysia, 2010) | Cross-sectional | Kelantan State (four districts bordering Thailand) | Patients with suspected chikungunya | 130 | 2009 (during the outbreak in southern Thailand) | IgM-ELISA , RT-PCR | [28] |
| 5 | Chua (Malaysia, 2010) | Hospital-based, cross-sectional | National Public Health Laboratory (NPHL), Kuala lumper | Suspected patients referred to the NPHL | 13,759 | Jan. 2006 to Dec.2009 | RT-PCR, virus isolation, IgM-ELISA | [29] |
| 6 | Wangchuk (Bhutan, 2013) | Hospital-based, cross-sectional | Southwestern districts | Patients with suspected chikungunya | 210 | July 2012  (during the outbreak) | RT-PCR, IgM-ELISA | [30] |
| 7 | Rezza (Yemen, 2014) | Hospital-based, outbreak investigation | Hodeidah | Patients with suspected chikungunya or dengue | 400 | 2012  (during the outbreak) | RT-PCR, IgM-ELISA | [31] |
| 8 | Kautz (Mexico, 2015) | Cross-sectional  (from patients seeking treatment and by house visits to identify patients) | Chiapas State | Patients with febrile arthralgia | 119 | Oct. to Dec. 2014  (during the outbreak) | Real-time RT-PCR, IgM-ELISA | [32] |
| 9 | Cigarroa-Toledo (Mexico, 2016) | Hospital-based, cross-sectional | Yucatán State in the southeast | Patients with febrile arthralgia | 51 | Aug. to Oct. 2015  (during the outbreak) | RT-PCR | [33] |
| 10 | Danis-Lozano (Mexico, 2017) | Hospital-based, cross-sectional | Chiapas State | Patients with suspected chikungunya | 112 | Nov. 2014 to Jun. 2015 | Real-time RT-PCR, IgM-ELISA | [34] |
| 11 | Carrera (Panama, 2017) | Surveillance of febrile patients | Multiple locations | Patients with suspected chikungunya | 413 | 2014–2015  (during the outbreak) | IgM-ELISA | [35] |
| 12 | Cunha (Brazil, 2017) | Hospital-based, cross-sectional | Sergipe State | Febrile patients presenting with an “arbovirus-like” infection with | 142 | February 2016 | RT-PCR, IgM-ELISA | [36] |

* Studies with small sample sizes were included if the suspected cases were reported during outbreak investigation within a well-defined timeframe. We used the random-effects model to meta-analyze the prevalence because of the heterogeneity to give higher weights and wider confidence intervals for the studies with smaller sample sizes; ECSA, East/Central/South African; ELISA, enzyme-linked immunosorbent assay; IFAT, indirect fluorescent antibody test; RNA, ribonucleic acid; RT-PCR, reverse transcriptase-polymerase chain reaction.
