# Supplementary material for: Multisectoral Approach to Address Chikungunya Outbreaks Driven by Human Mobility: A Systematic Review and Meta-Analysis
Source: J Infect Dis. 2020 Oct 29;222(Suppl 8):S709–16. doi: 10.1093/infdis/jiaa500 (PMC7594244; doi:10.1093/infdis/jiaa500)
Supplement: jiaa500_suppl_Supplementary_Table_3 [file jiaa500_suppl_supplementary_table_3.docx]

**Supplementary Table 3.** Checklist for appraisal of cross-sectional studies from countries/territories undergoing chikungunya outbreaks linked to human mobility with high-level evidence and included in prevalence pooling

| **Criterion** | **Items** | **Score** |
| --- | --- | --- |
| **1.** Description of study design | **A.** Cross-sectional or outbreak investigation of all suspected patients in a particular setting and within as a specified period reporting prevalence as an outcome | 2 |
|  | **B.** Unclear design or outbreak investigation or surveillance not reporting all suspected cases in a particular setting within a specified period | 1 |
| **2.** Study population (Categories: general population or suspected patients) | **A.** Specified to one category | 2 |
|  | **B.** Not clearly specified to one category | 1 |
| **3.** Study setting | **A.** Clearly described | 2 |
|  | **B.** Not clearly described | 1 |
| **4.** Sampling method* | A. Random (or inclusion of suspected cases reported during outbreak investigation within a specified period) | 2 |
|  | B. Convenience or unclearly described | 1 |
| **5.** Method(s) of IgG detection | A. Clearly described | 2 |
|  | B. Not clearly described | 1 |
| **6.** Method(s) of confirming recent infection | A. CHIKV RNA and/or culture (with or without IgM) | 2 |
|  | B. Not clearly mentioned | 1 |

* Studies with small sample sizes were included if the suspected cases were reported during outbreak investigation within a well-defined timeframe. We used the random-effects model to meta-analyze the prevalence because of the heterogeneity to give higher weights and wider confidence intervals for the studies with smaller sample sizes.

Method of scoring and assessment of risk of bias*

| **Calculation** (No. of criteria $\times$ score) | **Total score** | **Risk of bias** | **Quality of study** |
| --- | --- | --- | --- |
| (5 $\times$ 2) + (0 $\times$ 1) | 10 | Low | High |
| (4 $\times$ 2) + (1 $\times$ 1) | 9 | Low | High |
| (3 $\times$ 2) + (2 $\times$ 1) | 8 | Moderate | Moderate |
| (2 $\times$ 2 ) + (3 $\times$ 1) | 7 | Moderate | Moderate |
| (1 $\times$ 2 ) + (4 $\times$ 1) | 6 | High | Low |
| (0 $\times$ 2 ) + (5 $\times$ 1) | 5 | High | Low |

* Studies were assessed against five of the six criteria, where those for pooling the IgG seroprevalence were not assessed with Criterion #6 and those for pooling the prevalence of laboratory-confirmed recent infections were not assessed with Criterion #5.
